# Liraglutide Attenuates Multiorgan Oxidative, Astroglial, and Mitochondrial Stress Responses in Thioacetamide-Induced Hepatic Encephalopathy

**DOI:** 10.3390/ijms27177538

**Published:** 2026-08-23

**Authors:** Yasin Bilgin, Fatih Mehmet Sari, Betul Cicek, Engin Kurt, Mustafa Ozkaraca, Ali Gungor, Nezahat Kurt, Asli Ozbek Bilgin

**Affiliations:** 1 Department of Emergency Medicine, Faculty of Medicine, Erzincan Binali Yıldırım University, 24100 Erzincan, Türkiye; dr.ysn.blgn@gmail.com (Y.B.); drfmsari@gmail.com (F.M.S.); 2 Department of Physiology, Faculty of Medicine, Erzincan Binali Yıldırım University, 24100 Erzincan, Türkiye; 3 Department of Emergency Medicine, Mengücek Gazi Training and Research Hospital, 24100 Erzincan, Türkiye; engonge56@gmail.com; 4 Department of Pathology, Faculty of Veterinary Medicine, Cumhuriyet University, 58140 Sivas, Türkiye; mustafaozkaraca@cumhuriyet.edu.tr; 5 Laboratory Technician and Veterinary Health Program, Health Services Vocational School, Osmaniye Korkut Ata University, 80000 Osmaniye, Türkiye; aligungor@osmaniye.edu.tr; 6 Department of Biochemistry, Faculty of Medicine, Erzincan Binali Yıldırım University, 24100 Erzincan, Türkiye; nezahat.kurt@erzincan.edu.tr; 7 Department of Pharmacology, Faculty of Medicine, Erzincan Binali Yıldırım University, 24100 Erzincan, Türkiye

**Keywords:** hepatic encephalopathy, liraglutide, thioacetamide, intestine, liver, brain, oxidative stress, astroglial activation, mitochondrial homeostasis, VEGFA

## Abstract

Hepatic encephalopathy (HE) is a complex complication of liver failure involving oxidative stress, astroglial activation, mitochondrial dysfunction, and multiorgan disturbances along the gut–liver–brain axis. This study investigated the therapeutic effects of liraglutide (LIRA) in a thioacetamide (TAA)-induced rat model of HE. Thirty male Sprague–Dawley rats were assigned to control, TAA, and TAA+LIRA 100, 200, or 400 µg/kg groups. HE was induced with TAA (200 mg/kg, intraperitoneally) for three consecutive days, followed by subcutaneous LIRA treatment for 14 days. Serum biochemical parameters, tissue oxidative and inflammatory markers, histopathology, and Nrf2, MFN2, AKT1, mTOR, GFAP, and VEGFA immunoreactivity were evaluated. TAA caused marked hepatic and cerebral structural injury, increased serum and brain glutamine concentrations, disrupted redox homeostasis, elevated MPO activity, and increased cerebral GFAP and multiorgan VEGFA immunoreactivity. LIRA partially improved hepatic and cerebral histopathology, reduced glutamine disturbances, attenuated selected oxidative, nitrosative, and inflammatory alterations, and modulated Nrf2, MFN2, AKT1, mTOR, GFAP, and VEGFA immunoreactivity in a tissue- and dose-specific manner. The 200 µg/kg dose produced the most consistent overall improvement, whereas the 400 µg/kg dose was less favorable for several endpoints. LIRA may therefore alleviate multiorgan stress associated with experimental HE, although further studies are required to confirm functional and mechanistic relevance.

## 1. Introduction

Hepatic encephalopathy (HE) remains one of the most severe and clinically challenging complications of acute and chronic liver failure, characterized by a wide spectrum of neuropsychiatric abnormalities ranging from subtle cognitive impairment to coma. Beyond these manifestations, HE imposes a substantial clinical burden, associated with frequent emergency department visits, recurrent hospitalizations and readmissions, impaired quality of life, poor prognosis, and elevated mortality [1,2]. The pathogenesis of HE involves a complex interplay among hyperammonemia, systemic inflammation, immune dysregulation, and oxidative/nitrosative stress, collectively promoting neuroglial dysfunction and neurocognitive impairment [3,4].

Recent evidence has documented that HE is a multiorgan syndrome resulting from complex interactions among the liver, intestine, and brain, rather than an isolated cerebral disorder [3,4]. Mechanistically, gut dysbiosis and intestinal barrier dysfunction increase portal delivery of ammonia and proinflammatory products, whereas impaired hepatic clearance permits their systemic accumulation [3,5]. Together, these mediators disrupt blood–brain barrier integrity and trigger glutamine-driven mitochondrial dysfunction, oxidative/nitrosative stress, microglial activation, and neuroinflammation in the brain [6].

At the molecular level, oxidative burden contributes to HE pathophysiology through reactive oxygen species (ROS)-dependent lipid peroxidation (LPO) and damage to cellular macromolecules [4]. In this context, nuclear factor erythroid 2-related factor 2 (NRF2) is a key redox-sensitive transcriptional regulator that coordinates the antioxidant response by promoting the expression of cytoprotective and detoxifying genes, thereby mitigating oxidative stress [7]. Mitofusin-2 (MFN2) is an essential regulator for mitochondrial network integrity. Its impairment disrupts mitochondrial fusion, promotes fragmentation, and drives oxidative injury, inflammation, and neuronal loss [8]. Nrf2-associated antioxidant responses and MFN2-related mitochondrial dynamics have been implicated in HE, with their dysregulation potentially increasing cellular susceptibility to oxidative damage [7,9]. Additionally, the AKT/mTOR pathway functions as a redox-sensitive regulator of cellular survival and metabolic adaptation, and alterations in AKT/mTOR-associated responses have been reported in experimental HE [10,11].

Neurovascular dysfunction contributes to the pathophysiology of HE by impairing blood–brain barrier (BBB) integrity and facilitating inflammatory and oxidative injury within the neurovascular unit [6]. Vascular endothelial growth factor (VEGF) is a major angiogenic mediator that can increase BBB permeability under pathological conditions [12]. VEGFA may be produced by reactive astrocytes during inflammatory stress [12], contributes to angiogenic remodeling and fibrogenic progression in the liver [13], and has been implicated in inflammation-associated activation and remodeling of intestinal microvessels [14]. Importantly, increased cerebral and circulating VEGF levels have been reported in experimental acute liver failure, suggesting that dysregulated VEGF-related responses may contribute to neurovascular alterations and represent a potential therapeutic target [15].

In this regard, liraglutide (LIRA), a glucagon-like peptide-1 receptor (GLP-1R) agonist, is of particular interest because, beyond its glucose-lowering effects, it has been associated with modulation of oxidative stress, mitochondrial function, cellular energy metabolism, and inflammatory responses in experimental models [16]. LIRA has also been shown to attenuate hepatic injury and fibrosis in different experimental settings [17,18]. Beyond its hepatic effects, LIRA has been reported to exert neuroprotective actions through GLP-1R-related support of mitochondrial function and energy metabolism, together with modulation of cellular survival and autophagy-related stress responses [19,20]. Beyond these actions, LIRA may also modulate systemic immunometabolic crosstalk linking the liver, spleen, and brain [21,22,23]. Thioacetamide (TAA)-evoked hepatocellular damage can promote DAMP release and excessive ROS production, potentially activating splenic macrophages and monocytes through TLR4–NF-κB/NLRP3-associated signaling and thereby amplifying systemic cytokine and oxidative responses [24,25]. Through GLP-1R-dependent mechanisms, LIRA may prevent these inflammatory and redox pathways while improving mitochondrial function and AMPK–PGC-1α-related metabolic adaptation [21,26]. Reduced hepatic inflammatory output and systemic ROS/cytokine burden, together with GLP-1R-related cAMP–PKA–CREB signaling in the brain, may afterward restrict astroglial and mitochondrial stress [27]. Although splenic responses were not directly assessed, this liver–spleen–brain immunometabolic loop provides a plausible complementary framework for LIRA’s multiorgan stress-modulating effects in HE. However, the relevance of these pleiotropic effects to HE remains unclear, supporting the rationale for evaluating LIRA in this context. However, whether these pleiotropic effects extend to hepatic encephalopathy remains unclear, providing a rationale for evaluating LIRA in this context.

To address this therapeutic gap, the present study investigated the effects of LIRA in an experimental rat model of HE, with particular emphasis on interconnected pathological responses along the gut–liver–brain axis. We assessed serum and cerebral glutamine, circulating glucose concentrations, tissue oxidative and inflammatory alterations, hepatic and cerebral histopathology, and Nrf2, MFN2, AKT1, mTOR, GFAP, and VEGFA immunoreactivity. Intestinal VEGFA immunoreactivity was additionally evaluated as a potential marker of vascular stress within the gut component of this axis. We hypothesized that LIRA would attenuate hepatic and cerebral injury and modulate tissue-specific metabolic, redox, astroglial, mitochondrial, and vascular stress responses associated with HE.

## 2. Results

### 2.1. Effects of LIRA on Serum Aminotransferases and Glutamine Dysregulation in TAA-Induced Hepatic Encephalopathy

**Figure 1:**
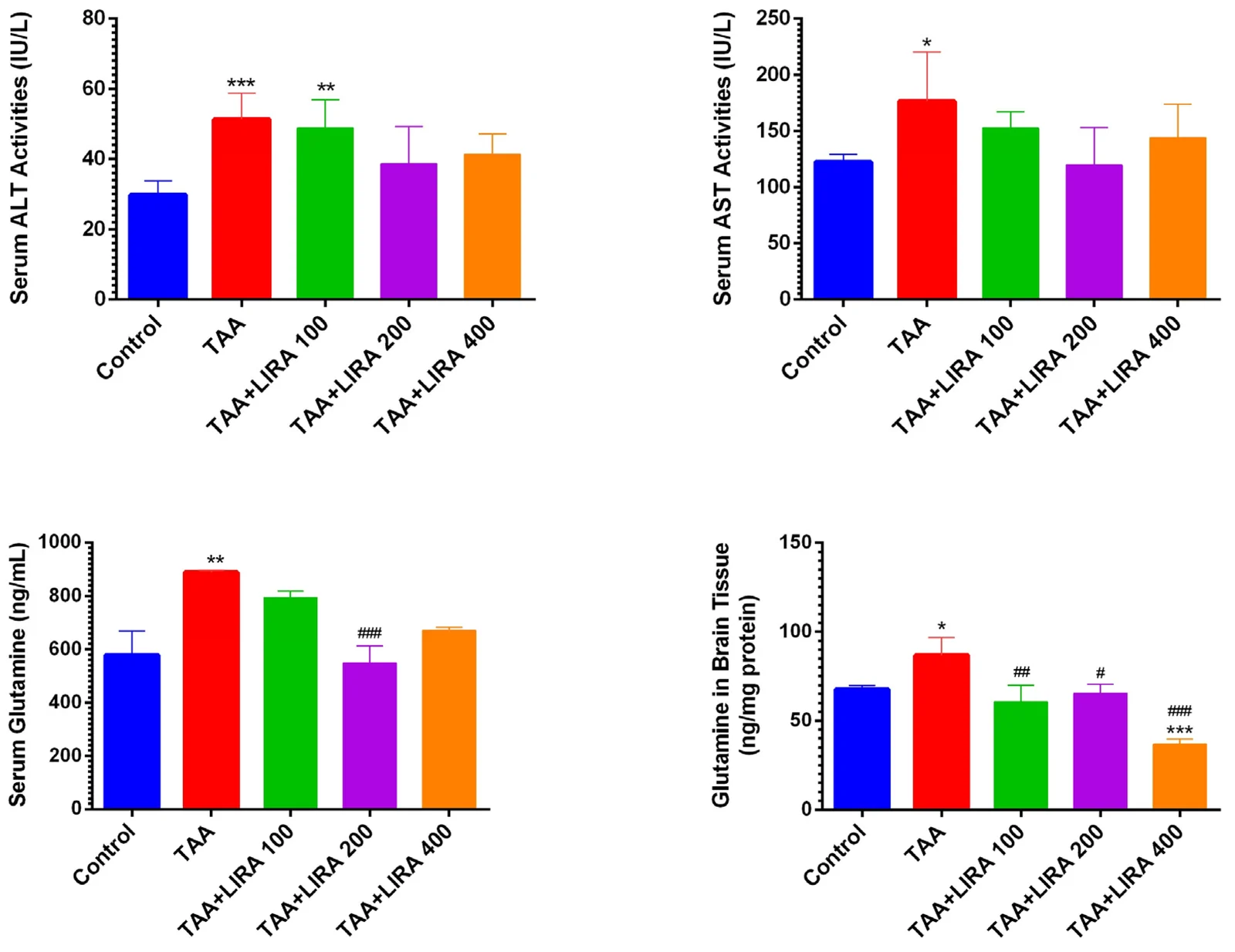
The effects of liraglutide (LIRA) on serum aminotransferase activities and glutamine concentrations in rats with thioacetamide-induced hepatic encephalopathy. Serum ALT and AST activities and serum and brain glutamine concentrations were evaluated in the control, TAA, TAA+LIRA 100, TAA+LIRA 200, and TAA+LIRA 400 µg/kg groups. ALT data were analyzed using one-way ANOVA followed by Tukey’s HSD post hoc test, whereas AST data were analyzed using the Kruskal–Wallis test followed by Bonferroni-adjusted pairwise comparisons. Brain glutamine data were analyzed using Welch’s one-way ANOVA followed by the Games–Howell post hoc test, and serum glutamine data were analyzed using the Kruskal–Wallis test followed by Bonferroni-adjusted pairwise comparisons. ALT and brain glutamine data are presented as the mean ± SD, whereas AST and serum glutamine data are presented as median with interquartile range. Data were obtained from six rats per group. * *p* < 0.05, ** *p* < 0.01, and *** *p* < 0.001 versus the control group; # *p* < 0.05, ## *p* < 0.01, and ### *p* < 0.001 versus the TAA group.

TAA administration significantly increased serum ALT and AST activities compared with the control group, supporting the induction of hepatic injury. LIRA treatment produced a non-linear pattern of change in serum transaminase activities. Among the tested doses, LIRA at 200 µg/kg was associated with the greatest numerical reduction in both ALT and AST activities relative to the TAA group. However, these reductions did not reach statistical significance after multiple-comparison adjustment. ALT and AST activities in the TAA+LIRA 200 µg/kg group were no longer significantly different from those in the control group, suggesting a tendency toward biochemical normalization rather than a statistically confirmed treatment effect (Figure 1).

Consistent with the hepatic injury pattern, TAA significantly disrupted glutamine homeostasis, increasing both serum and brain glutamine levels compared with the control group and supporting the development of HE-like disturbances in ammonia–glutamine metabolism. In brain tissue, all LIRA doses significantly reduced the TAA-induced increase in glutamine. The 100 and 200 µg/kg doses restored brain glutamine levels to values comparable with the control group, whereas the 400 µg/kg dose reduced brain glutamine to values significantly below those of the control and lower-dose treatment groups. In serum, the most consistent restorative response was observed with LIRA at 200 µg/kg, which significantly reduced glutamine levels compared with the TAA and TAA+LIRA 100 µg/kg groups and restored them to a level comparable with the control group. Although LIRA at 400 µg/kg also tended to reduce serum glutamine relative to TAA, this difference did not remain statistically significant after adjustment for multiple comparisons. Overall, these findings indicate that LIRA ameliorated TAA-induced disturbances in glutamine metabolism, with 200 µg/kg providing the most consistent normalization across both serum and brain compartments (Figure 1).

### 2.2. LIRA Ameliorates TAA-Induced Hepatic and Cerebral Structural Alterations

**Figure 2:**
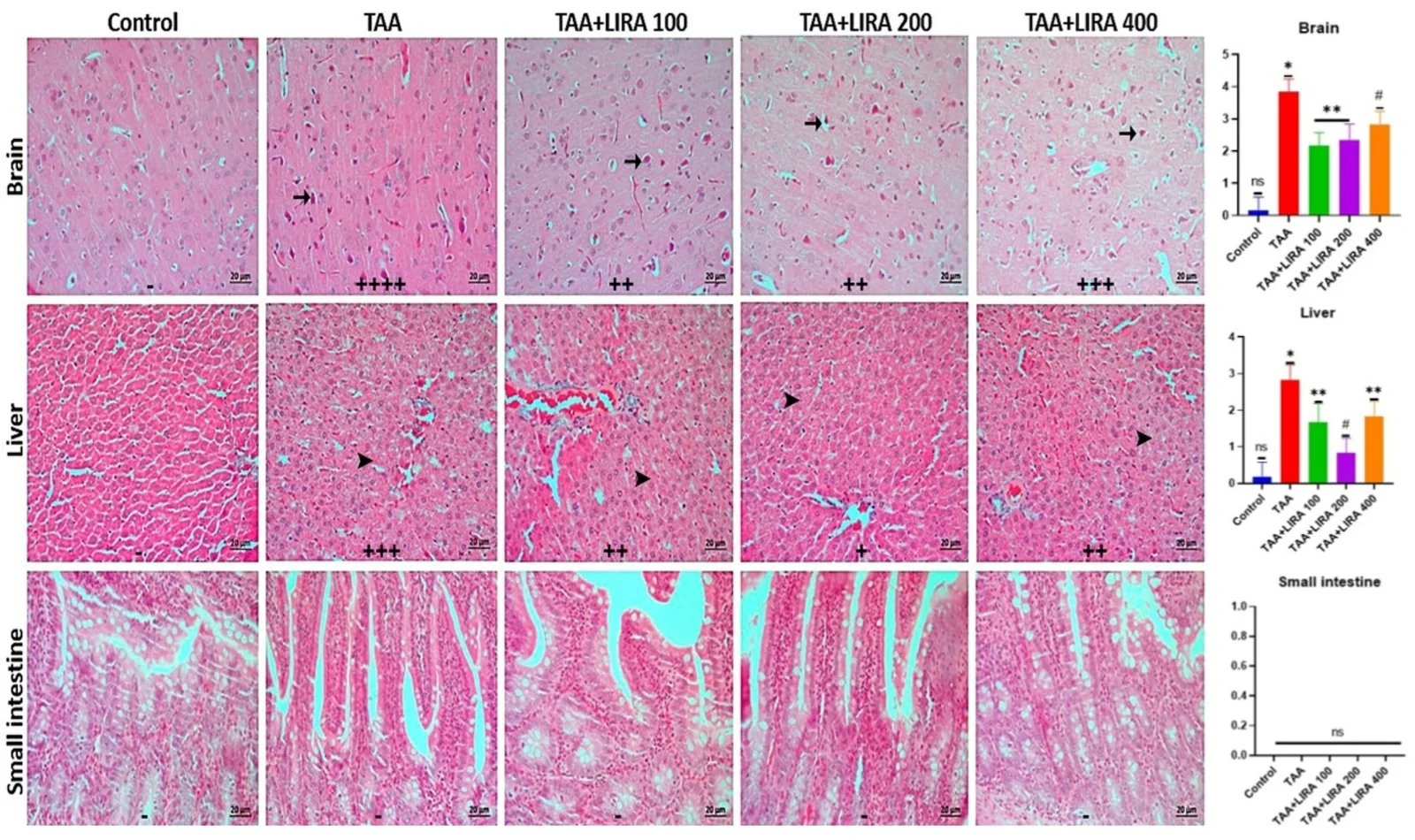
The effect of liraglutide (LIRA) treatment on thioacetamide (TAA)-induced histopathological alterations in hepatic, small intestinal and cerebral tissues. Representative hematoxylin and eosin-stained tissue sections and semiquantitative histopathological scores from the experimental groups (*n* = 6 rats per group). Low-magnification images were obtained at ×100 with a scale bar of 100 µm, and high-magnification images were obtained at ×400 with a scale bar of 20 µm. Liver sections from the control group showed preserved hepatic architecture, whereas TAA administration induced severe hepatocellular necrosis and marked structural disorganization. LIRA treatment attenuated hepatic injury in a non-linear dose-related manner. Moderate hepatocellular necrosis was observed in the TAA+LIRA 100 µg/kg and TAA+LIRA 400 µg/kg groups, whereas the TAA+LIRA 200 µg/kg group exhibited only mild necrotic changes and the most favorable hepatic recovery. Brain sections from the control group showed preserved neuronal morphology. TAA administration produced severe neuronal injury characterized by prominent pyknotic and degenerative neuronal changes. LIRA treatment reduced cerebral damage, with more pronounced improvement in the TAA+LIRA 100 µg/kg and TAA+LIRA 200 µg/kg groups, in which neuronal alterations were moderate. Although LIRA at 400 µg/kg attenuated neuronal injury compared with TAA alone, severe degenerative changes remained evident. Small intestinal sections showed no evident pathological alterations in any experimental group. Arrows indicate degenerative neurons in brain tissue and necrotic areas in liver tissue; plus signs indicate the corresponding histopathological changes. Hematoxylin and eosin (H&E)-stained sections were semiquantitatively evaluated for necrotic and degenerative changes and scored as absent (−), mild (+), moderate (++), severe (+++), or very severe (++++). Semiquantitative scoring demonstrated significant intergroup differences in hepatic and cerebral tissues, whereas no significant difference was detected in small intestinal tissue. Data are presented as the median and interquartile range. Group differences were evaluated using the Kruskal–Wallis test. Symbols (*, **, #) indicate statistically significant differences between groups (*p* < 0.05); ns, not significant.

TAA markedly disrupted hepatic architecture, as evidenced by severe hepatocellular necrosis and extensive structural disorganization in liver sections. These histopathological alterations confirmed substantial TAA-induced hepatic damage. LIRA treatment mitigated these degenerative alterations in a non-linear dose-related manner. The TAA+LIRA 100 µg/kg and TAA+LIRA 400 µg/kg groups presented moderate hepatocellular necrosis, reflecting partial histological improvement compared with the TAA group. Notably, the TAA+LIRA 200 µg/kg group displayed only mild necrotic changes, demonstrating the most pronounced restoration of hepatic tissue integrity among the treatment groups. These findings suggest that LIRA attenuated TAA-evoked hepatic structural injury, with 200 µg/kg representing the most effective dose for histopathological improvement (Figure 2).

TAA administration also markedly disrupted cerebral architecture. Brain sections from the TAA group exhibited severe neuronal injury characterized by prominent pyknotic and degenerative neuronal changes. LIRA treatment attenuated TAA-induced cerebral damage, with the greatest histopathological improvement observed in the TAA+LIRA 100 µg/kg and TAA+LIRA 200 µg/kg groups, in which neuronal alterations were reduced to a moderate degree. Although LIRA at 400 µg/kg also decreased neuronal injury compared with the TAA group, brain sections from this group continued to exhibit severe pyknotic and degenerative neuronal changes. Collectively, these findings demonstrate that LIRA ameliorated TAA-induced hepatic and cerebral structural injury, although its protective effects were not linearly dose-dependent.

In contrast to these findings, microscopic examination of small intestinal sections revealed no evident pathological alterations in any experimental group. Consistently, semiquantitative histopathological scores did not differ significantly among the groups, suggesting that TAA-induced tissue injury in this experimental model was primarily evident in the liver and brain rather than in the small intestine.

### 2.3. LIRA Does Not Alter Fasting Blood Glucose Concentrations in TAA-Induced HE

**Figure 3:**
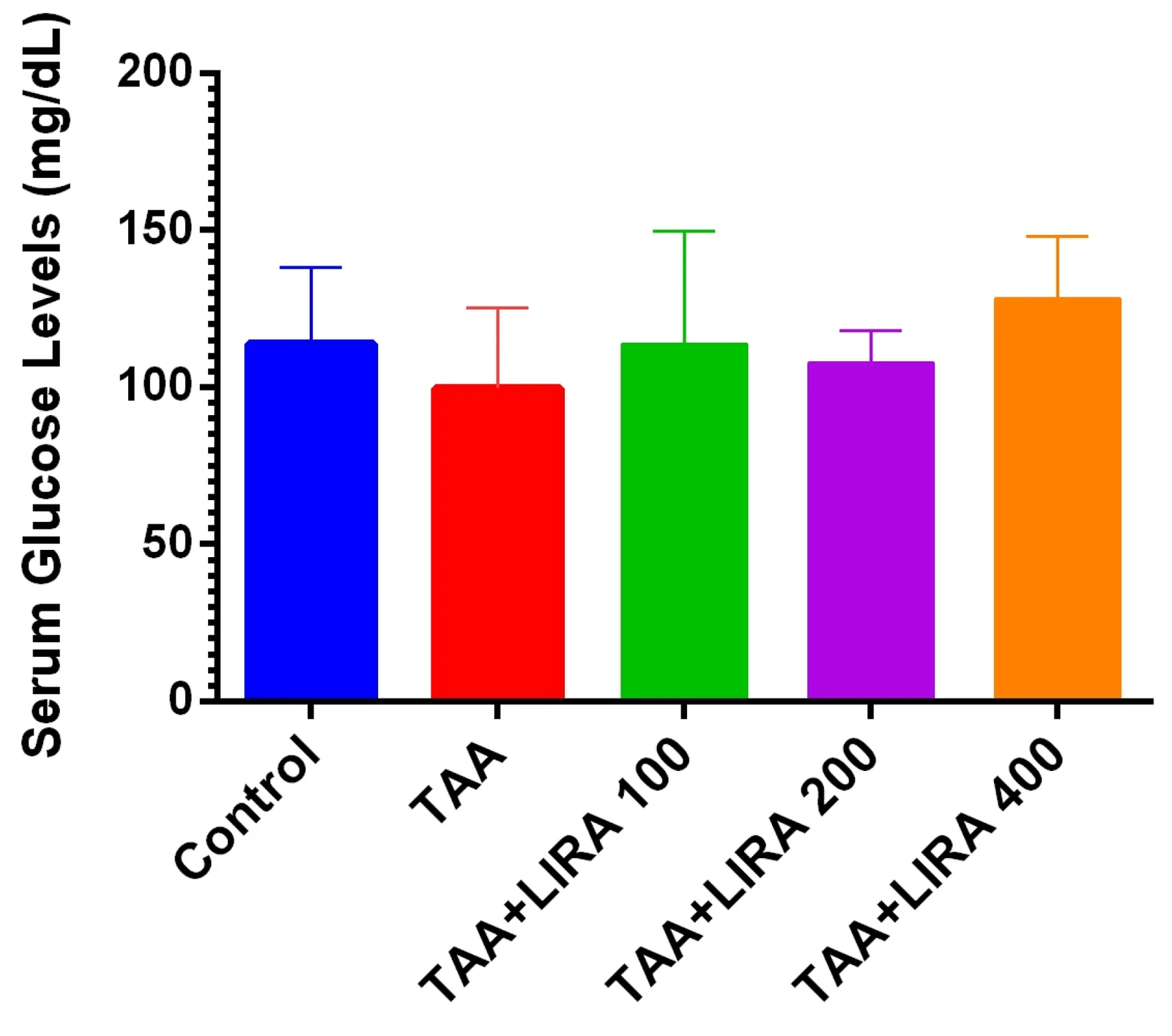
The effects of liraglutide (LIRA) on fasting blood glucose concentrations in thioacetamide (TAA)-intoxicated rats. Blood glucose concentrations were measured in the control, TAA, TAA+LIRA 100, TAA+LIRA 200, and TAA+LIRA 400 µg/kg groups to evaluate systemic glucose homeostasis. No statistically significant differences were observed among the groups. Data are presented as the mean ± SD, with *n* = 6 rats per group.

Fasting blood glucose concentrations were evaluated to determine whether TAA-induced HE or LIRA treatment affected systemic glucose homeostasis. No statistically significant differences in blood glucose concentrations were determined among all groups. These outcomes indicate that neither TAA administration nor LIRA treatment generated notable changes in circulating glucose concentrations under the experimental conditions (Figure 3).

### 2.4. LIRA Ameliorates TAA-Evoked Oxidative Stress in Association with Increased Nrf2 Immunoreactivity

**Figure 4:**
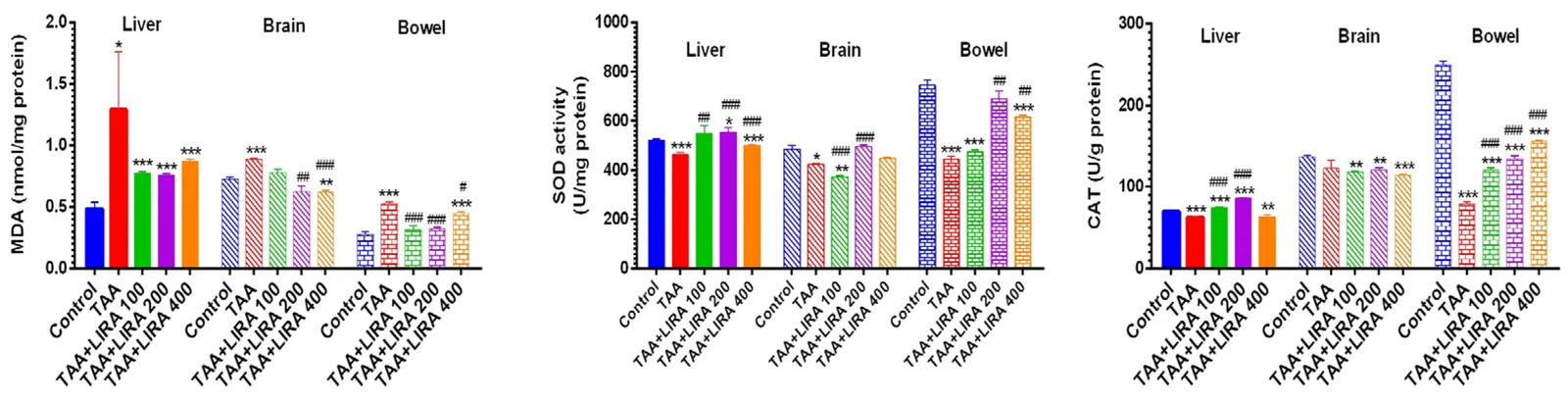
The effects of liraglutide (LIRA) on oxidative stress parameters in liver, brain, and small intestinal tissues of rats with thioacetamide-induced hepatic encephalopathy. MDA levels, SOD activity, and CAT activity were evaluated in the control, TAA, TAA+LIRA 100, TAA+LIRA 200, and TAA+LIRA 400 µg/kg groups. Data are presented as the mean ± SD (*n* = 6 per group). Variables with unequal variances were analyzed using Welch’s one-way ANOVA followed by the Games–Howell post hoc test, whereas variables meeting the homogeneity of variance assumption were analyzed using one-way ANOVA followed by Tukey’s HSD test. * *p* < 0.05, ** *p* < 0.01, and *** *p* < 0.001 versus the control group; # *p* < 0.05, ## *p* < 0.01, and ### *p* < 0.001 versus the TAA group.

TAA administration markedly disrupted the oxidant–antioxidant balance in hepatic, cerebral, and intestinal tissues. Compared with the control group, TAA significantly increased MDA levels in all three tissues, indicating enhanced lipid peroxidation. TAA also reduced SOD activity in the liver, brain, and intestine and decreased CAT activity in the liver and intestine, whereas cerebral CAT activity was not significantly altered. LIRA exerted tissue, marker, and dose-dependent effects on these oxidative changes. In the liver, all LIRA doses significantly increased SOD activity, while the 100 and 200 µg/kg doses enhanced CAT activity compared with the TAA group. However, none of the LIRA doses significantly reduced hepatic MDA levels relative to TAA. In the brain, LIRA at 200 µg/kg restored SOD activity to the control range and significantly reduced MDA levels, whereas the 400 µg/kg dose produced a greater reduction in MDA but did not significantly improve SOD relative to TAA. None of the LIRA doses significantly increased cerebral CAT activity compared with the TAA group. In the intestine, the 100 and 200 µg/kg doses normalized MDA levels, while the 200 µg/kg dose restored SOD activity to the control range. All LIRA doses increased intestinal CAT activity compared with TAA, with the greatest increase observed at 400 µg/kg, although values remained below those of the control group. Overall, LIRA attenuated several TAA-induced oxidative alterations, with the 200 µg/kg dose providing the most consistent overall improvement, while the response pattern remained non-linear and dependent on tissue and biochemical marker evaluated (Figure 4).

**Figure 5:**
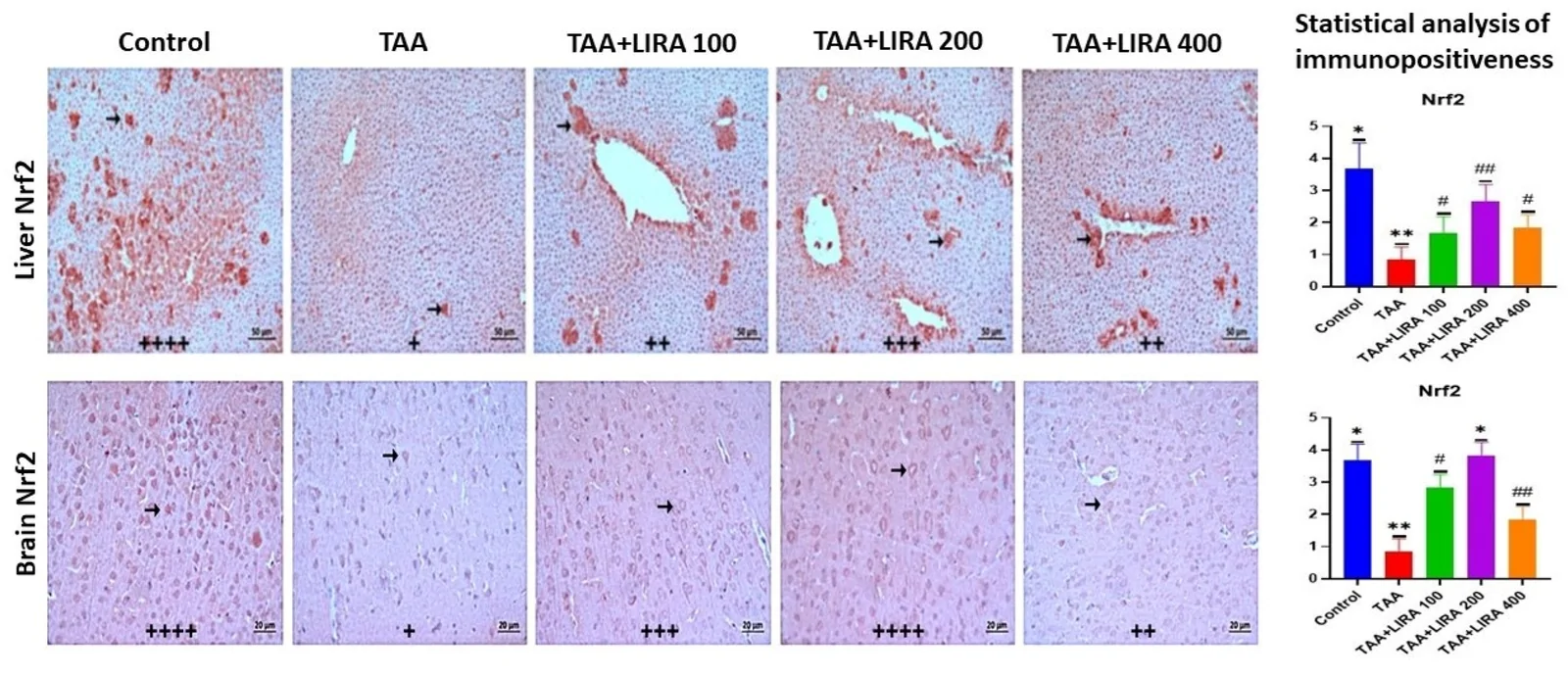
The effects of liraglutide (LIRA) on Nrf2 immunoreactivity in liver and brain tissues of rats with thioacetamide-induced hepatic encephalopathy. Representative immunohistochemical images and semiquantitative analyses of Nrf2 immunoreactivity are shown for the control, TAA, TAA+LIRA 100, TAA+LIRA 200, and TAA+LIRA 400 µg/kg groups. TAA reduced Nrf2 immunoreactivity in both liver and brain tissues, whereas LIRA treatment increased Nrf2 immunoreactivity, with the most pronounced response observed at 200 µg/kg. Arrows indicate positive immunostaining. Nrf2 immunoreactivity was graded semiquantitatively as mild (+), moderate (++), severe (+++), or very severe (++++). DAB–hematoxylin staining; scale bars: 50 µm for liver and 20 µm for brain. Data are presented as the median with the interquartile range (*n* = 6 per group). Group differences were evaluated using the Kruskal–Wallis test. Symbols (*, **, #, ##) indicate statistically significant differences between groups (*p* < 0.05).

To assess whether TAA-induced hepatic encephalopathy was associated with the suppression of endogenous antioxidant defense responses, Nrf2 immunoreactivity was evaluated in liver and brain tissues. TAA markedly reduced Nrf2 immunoreactivity in both tissues compared with the control group. LIRA treatment reversed this reduction to varying degrees. In the liver, Nrf2 immunoreactivity increased in all LIRA-treated groups relative to TAA, with the most pronounced restoration observed at 200 µg/kg. A similar pattern was detected in the brain, where the 200 µg/kg dose produced the strongest restoration of Nrf2 immunoreactivity, while the responses to the 100 and 400 µg/kg doses were less marked. These findings indicate that the antioxidant effects of LIRA occurred in association with increased Nrf2 immunoreactivity, particularly at 200 µg/kg (Figure 5).

### 2.5. LIRA Differentially Modulates Tissue MPO Activity and NO Metabolite Levels

**Figure 6:**
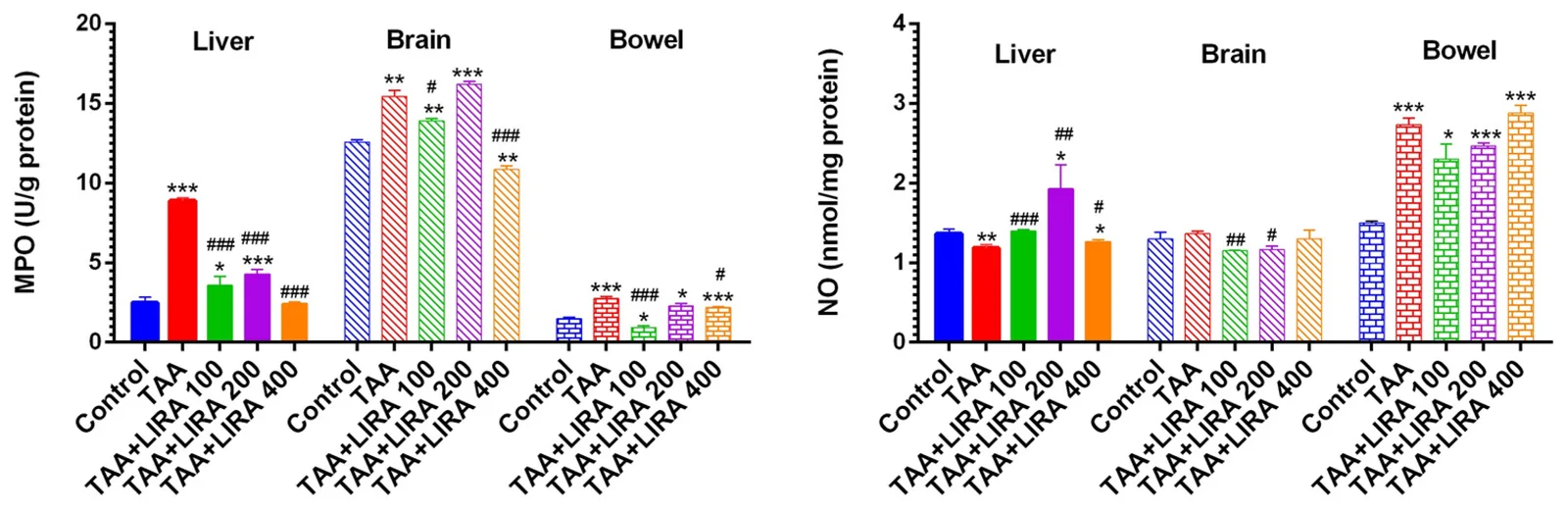
The effects of liraglutide (LIRA) on MPO activity and NO metabolite levels in the liver, brain, and small intestine of rats with TAA-induced hepatic encephalopathy. MPO activity and NO metabolite levels were determined in the control, TAA, TAA+LIRA 100, TAA+LIRA 200, and TAA+LIRA 400 µg/kg groups. LIRA exerted tissue-, marker-, and dose-dependent effects on TAA-induced inflammatory and nitrosative stress markers. Data are expressed as the mean ± SD, with *n* = 6 animals per group Statistical analyses were performed using Welch’s one-way ANOVA followed by the Games–Howell post hoc test. * *p* < 0.05, ** *p* < 0.01, and *** *p* < 0.001 versus the control group; # *p* < 0.05, ## *p* < 0.01, and ### *p* < 0.001 versus the TAA group.

TAA administration altered MPO activity and NO metabolite levels in a tissue-dependent manner. In the liver, TAA significantly increased MPO activity while reducing NO levels compared with the control group. All LIRA doses significantly decreased hepatic MPO activity and increased NO metabolite levels relative to the TAA group. The 400 µg/kg dose restored hepatic MPO activity to values comparable with those of the control group, whereas the 100 µg/kg dose restored hepatic NO metabolite levels to the control range. In brain tissue, TAA significantly increased MPO activity but did not significantly alter NO metabolite levels compared with the control group. LIRA at 100 and 400 µg/kg significantly reduced cerebral MPO activity relative to the TAA group, whereas the 200 µg/kg dose did not differ significantly from the TAA group. Cerebral NO metabolite levels were lower in the 100 and 200 µg/kg groups than in the TAA group, although none of the LIRA -treated groups differed significantly from the control group. In small intestinal tissue, TAA significantly increased both MPO activity and NO metabolite levels. LIRA at 100 and 400 µg/kg significantly reduced intestinal MPO activity, whereas the 200 µg/kg dose did not produce a significant change. None of the LIRA doses significantly reversed the TAA-induced increase in intestinal NO metabolite levels. Overall, LIRA exerted tissue-, marker-, and dose-dependent effects on inflammatory and nitrosative stress, without a uniform linear dose–response pattern (Figure 6).

### 2.6. LIRA Attenuates TAA-Induced Astroglial Activation in Cerebral Tissue

**Figure 7:**
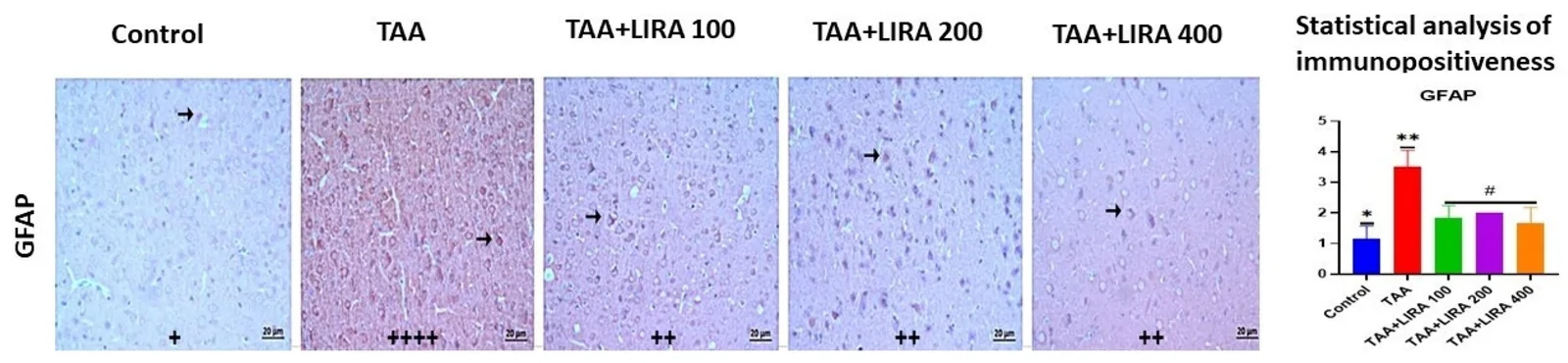
The effect of liraglutide (LIRA) treatment on glial fibrillary acidic protein immunoreactivity in the cerebral tissue of rats with thioacetamide-induced hepatic encephalopathy. Representative immunohistochemical images of glial fibrillary acidic protein (GFAP) immunoreactivity in cerebral tissue are shown for the control, TAA, TAA+LIRA 100, TAA+LIRA 200, and TAA+LIRA 400 µg/kg groups (*n* = 6 per group). The control group exhibited weak basal GFAP immunoreactivity. TAA administration markedly increased GFAP immunoreactivity, indicating pronounced astroglial activation. LIRA treatment at all tested doses reduced GFAP immunoreactivity relative to the TAA group, with moderate staining observed in all LIRA-treated groups. Arrows indicate GFAP-positive immunostaining. Low magnification: ×100, scale bar = 100 µm; high magnification: ×400, scale bar = 25 µm. The accompanying graph presents the semiquantitative analysis of GFAP immunoreactivity, scored semiquantitatively as mild (+), moderate (++), or very severe (++++). Data are presented as the median and interquartile range, with *n* = 6 per group. Statistical comparisons were performed using the Kruskal–Wallis test. *, **, # indicate statistically significant differences between the indicated groups (*p* < 0.05).

Given the central role of astrocytic activation in HE-related cerebral dysfunction, GFAP immunoreactivity was evaluated as a marker of reactive astrogliosis. TAA administration markedly increased GFAP immunoreactivity compared with the control group, indicating pronounced astroglial activation. LIRA treatment reduced TAA-induced GFAP immunoreactivity in all treated groups, suggesting attenuation of reactive astrogliosis. The magnitude of this reduction was broadly comparable among the LIRA-treated groups, with no clear linear dose–response relationship. Overall, these findings indicate that LIRA attenuated TAA-associated astroglial activation in cerebral tissue and may thereby contribute to the amelioration in HE-related neuroinflammatory alterations (Figure 7).

### 2.7. LIRA Differentially Modulates MFN2, AKT1, and mTOR Immunoreactivity in Hepatic and Cerebral Tissues

To investigate whether the effects of LIRA were associated with MFN2-related mitochondrial responses and AKT1/mTOR-related cellular responses along the liver–brain axis, MFN2, AKT1, and mTOR immunoreactivity was evaluated in hepatic and cerebral tissues. TAA administration reduced MFN2 immunoreactivity in both tissues compared with the respective control groups, indicating an alteration in MFN2-associated mitochondrial responses. In the liver, MFN2 immunoreactivity was markedly increased in the TAA+LIRA 100 and TAA+ LIRA 200 µg/kg groups, reaching levels comparable with those of the control group, whereas the 400 µg/kg dose showed only weak immunoreactivity. In cerebral tissue, the 200 µg/kg dose restored MFN2 immunoreactivity to the control range, while weak staining persisted in the 100 and 400 µg/kg groups.

**Figure 8:**
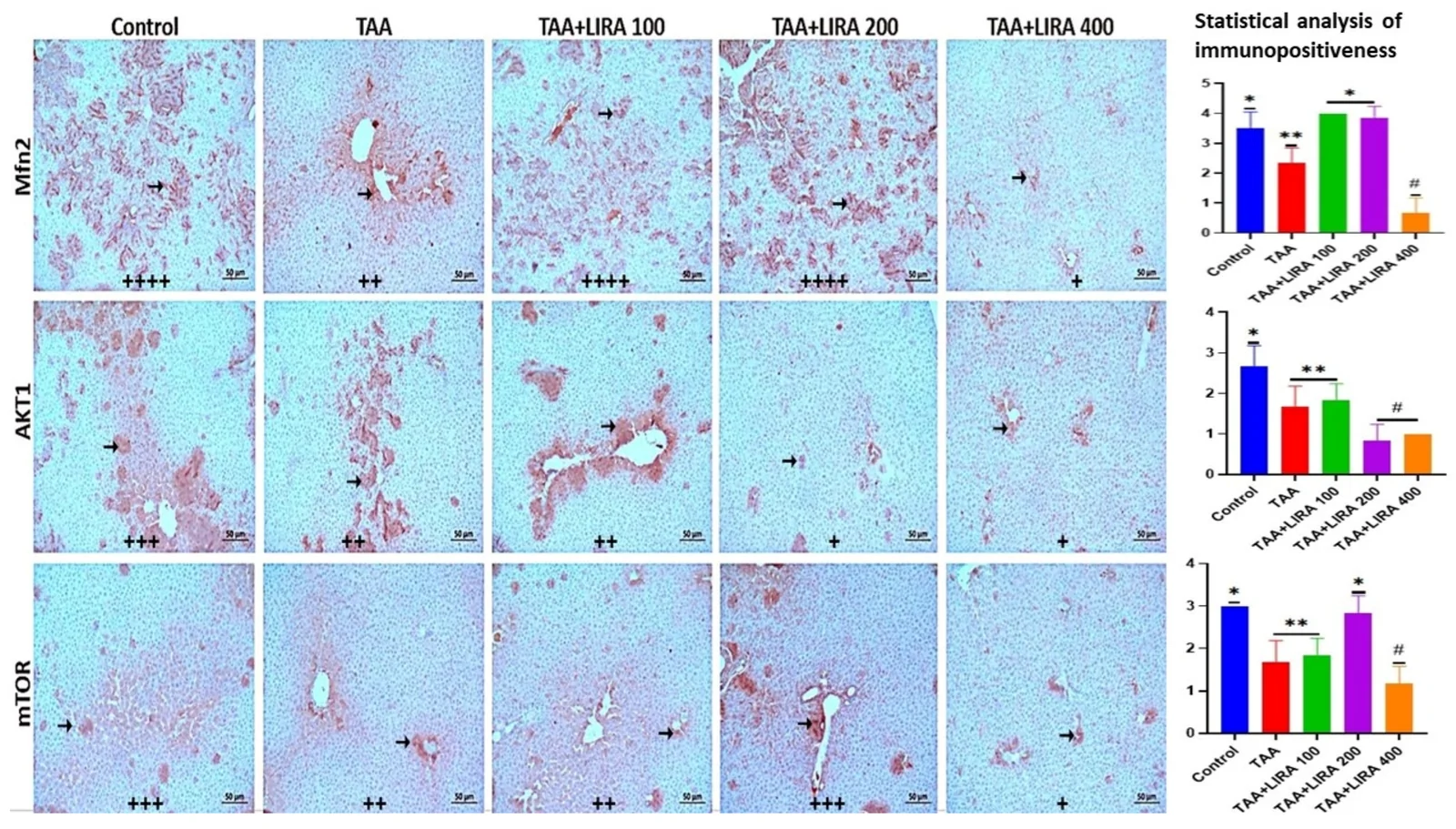
The effects of liraglutide (LIRA) on hepatic MFN2, AKT1, and mTOR immunoreactivity in rats with TAA-induced hepatic encephalopathy. Representative immunohistochemical images and semiquantitative analyses of MFN2, AKT1, and mTOR immunoreactivity are shown for the control, TAA, TAA+LIRA 100, TAA+LIRA 200, and TAA+LIRA 400 µg/kg groups. TAA reduced hepatic MFN2 and mTOR immunoreactivity compared with the control group. MFN2 immunoreactivity was most prominent in the TAA+LIRA 100 and TAA+LIRA 200 µg/kg groups, whereas weak staining was observed in the TAA+LIRA 400 µg/kg group. Hepatic mTOR immunoreactivity was most clearly restored by LIRA 200 µg/kg. In contrast, hepatic AKT1 immunoreactivity remained moderate in the TAA+LIRA 100 µg/kg group and mild in the TAA+LIRA 200 and TAA+LIRA 400 µg/kg groups. Arrows indicate positive immunostaining. Immunoreactivity was scored semiquantitatively as semiquantitatively as mild (+), moderate (++), severe (+++), or very severe (++++). DAB–hematoxylin staining; scale bar = 50 µm. Statistical comparisons were performed using the Kruskal–Wallis test. Symbols (*, **, #) indicate statistically significant differences between groups (*p* < 0.05).

**Figure 9:**
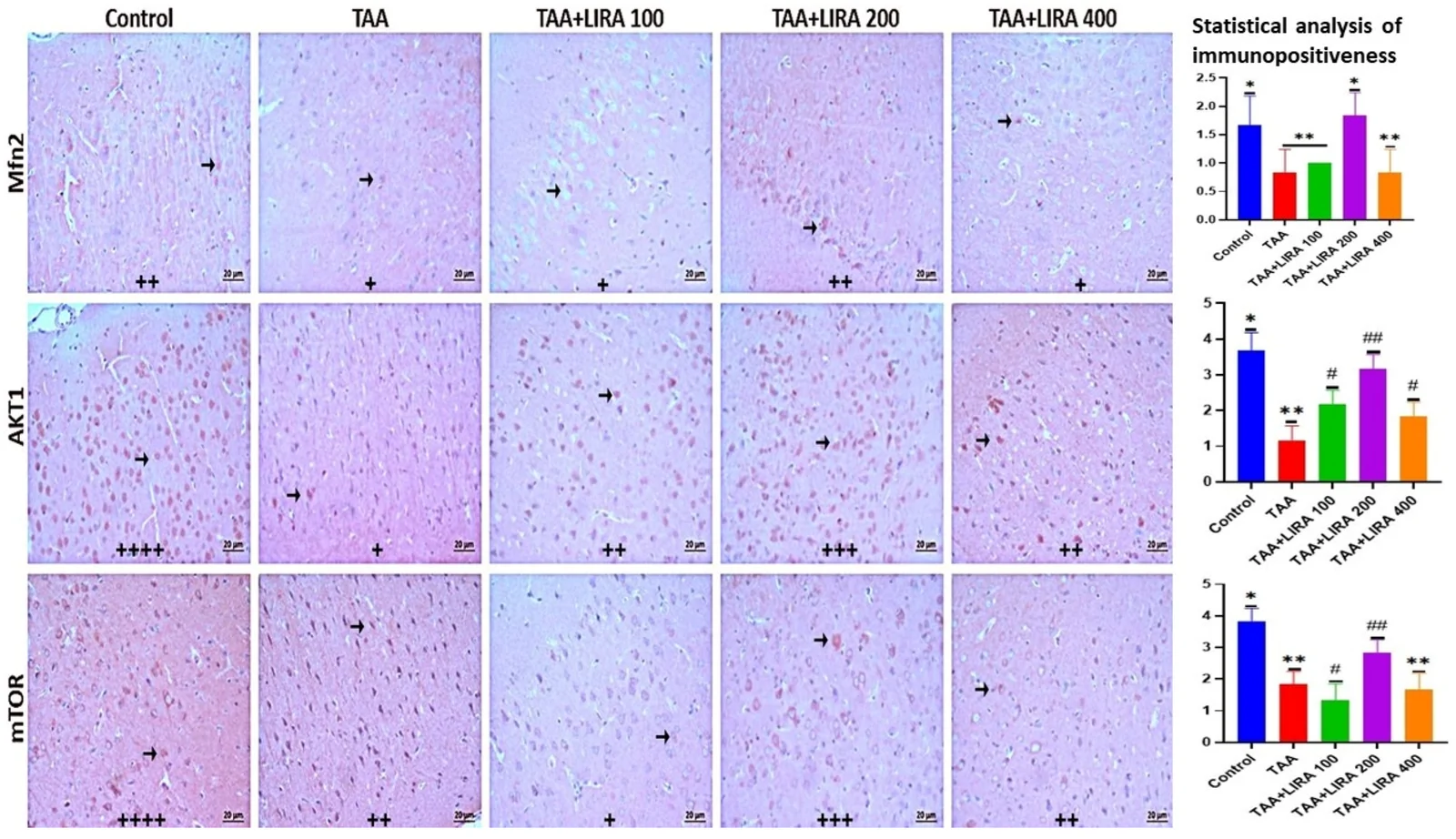
The effects of liraglutide (LIRA) on cerebral MFN2, AKT1, and mTOR immunoreactivity in rats with TAA-induced hepatic encephalopathy. Representative immunohistochemical images and semiquantitative analyses of MFN2, AKT1, and mTOR immunoreactivity are shown for the control, TAA, TAA+LIRA 100, TAA+LIRA 200, and TAA+LIRA 400 µg/kg groups (*n* = 6 per group). TAA reduced cerebral MFN2 and mTOR immunoreactivity compared with the control group. LIRA at 200 µg/kg produced the most pronounced restoration of all three markers, with MFN2 immunoreactivity returning to the control range and strong AKT1 and mTOR staining being observed. MFN2 immunoreactivity was most prominent in the TAA+LIRA 100 and TAA+LIRA 400 µg/kg groups. AKT1 immunoreactivity was moderate in the 100 and 400 µg/kg groups, whereas mTOR immunoreactivity was mild at 100 µg/kg and moderate at 400 µg/kg. Arrows indicate positive immunostaining. Immunoreactivity was scored semiquantitatively as mild (+), moderate (++), severe (+++), or very severe (++++). DAB–hematoxylin staining; scale bar = 20 µm. Statistical comparisons were performed using the Kruskal–Wallis test. Symbols (*, **, #, ##) indicate statistically significant differences between groups (*p* < 0.05).

TAA administration also reduced AKT1 and mTOR immunoreactivity in the brain. LIRA partially reversed these alterations, with the most pronounced cerebral response observed at 200 µg/kg. Cerebral AKT1 and mTOR immunoreactivity was severe in the TAA+LIRA 200 µg/kg group, whereas moderate AKT1 immunoreactivity was observed in the 100 and 400 µg/kg groups and moderate mTOR immunoreactivity in the 400 µg/kg group. In the liver, however, the response pattern differed. Hepatic mTOR immunoreactivity was restored most clearly by the 200 µg/kg dose, reaching a level comparable with the control group, whereas the 100 µg/kg dose remained moderate and the 400 µg/kg dose was weak. In contrast, hepatic AKT1 immunoreactivity was not restored by LIRA; it remained moderate at 100 µg/kg and was weak at 200 and 400 µg/kg. Overall, LIRA exerted tissue, marker, and dose-dependent effects on MFN2 and AKT1/mTOR-related immunoreactivity, with 200 µg/kg showing the most consistent response in cerebral tissue and for hepatic mTOR, but not for hepatic AKT1 (Figure 8 and Figure 9).

### 2.8. LIRA Modulates TAA-Induced VEGFA Immunoreactivity in Hepatic, Small Intestinal, and Cerebral Tissues

TAA administration markedly increased VEGFA immunoreactivity in hepatic, cerebral, and small intestinal tissues compared with the control group, indicating enhanced vascular and angiogenic stress responses. In the liver, VEGFA immunoreactivity increased from mild in the control group to severe in the TAA group. LIRA treatment at 100, 200, and 400 µg/kg reduced hepatic VEGFA immunoreactivity to a moderate level, indicating attenuation of the TAA-induced response at all tested doses.

**Figure 10:**
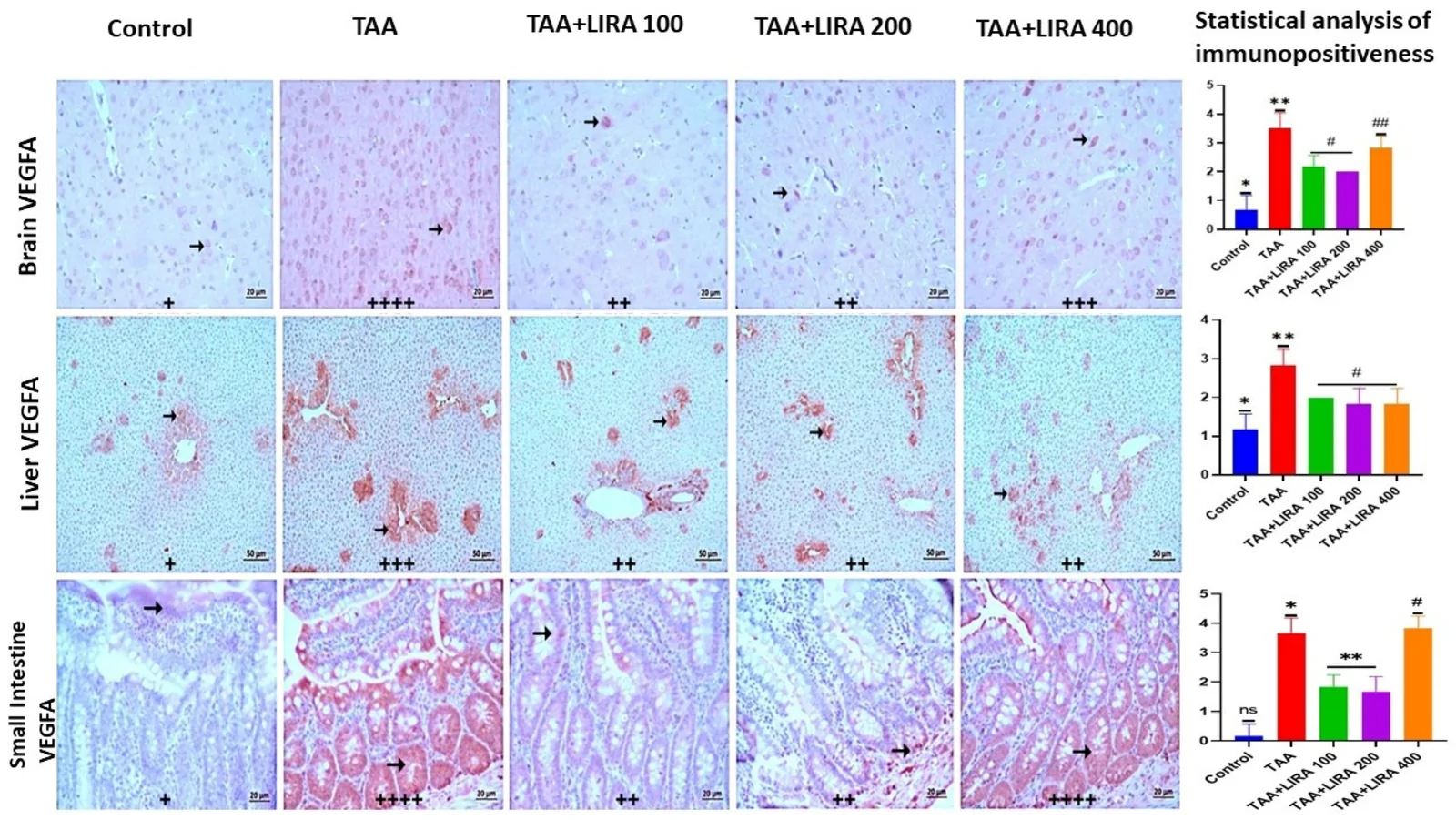
The effects of liraglutide (LIRA) on VEGFA immunoreactivity in cerebral, hepatic, and small intestinal tissues of rats with TAA-induced hepatic encephalopathy. Representative immunohistochemical images and semiquantitative analyses of vascular endothelial growth factor A (VEGFA) immunoreactivity is presented for the control, TAA, TAA+LIRA 100, TAA+LIRA 200, and TAA+LIRA 400 µg/kg groups (*n* = 6 per group). TAA administration markedly increased VEGFA immunoreactivity in all three tissues. In the liver, all LIRA doses reduced VEGFA immunoreactivity from severe to moderate levels. In cerebral tissue, the 100 and 200 µg/kg doses reduced VEGFA immunoreactivity to moderate levels, whereas staining remained severe in the 400 µg/kg group. In the small intestine, the 100 and 200 µg/kg doses similarly reduced VEGFA immunoreactivity to moderate levels, while the 400 µg/kg group retained very severe staining comparable in intensity with the TAA group. Arrows indicate positive VEGFA immunostaining. Immunoreactivity was scored semiquantitatively as mild (+), moderate (++), severe (+++), or very severe (++++). DAB–hematoxylin staining; scale bars = 20 µm for brain and small intestine and 50 µm for liver. Statistical comparisons were performed using the Kruskal–Wallis test. Symbols (*, **, #, ##) indicate statistically significant differences between groups (*p* < 0.05), ns, not significant.

In cerebral tissue, VEGFA immunoreactivity was mild in the control group and very severe in the TAA group. Treatment with LIRA at 100 and 200 µg/kg reduced VEGFA immunoreactivity to a moderate level, whereas the 400 µg/kg dose produced a less pronounced response, with staining remaining severe. Similarly, small intestinal VEGFA immunoreactivity was weak to mild in the control group and very severe following TAA administration. The 100 and 200 µg/kg LIRA doses reduced intestinal VEGFA immunoreactivity to a moderate level, while the 400 µg/kg dose failed to produce a comparable reduction and VEGFA staining remained very severe. Overall, LIRA modulated TAA-induced VEGFA dysregulation in a tissue- and dose-dependent manner, with the 100 and 200 µg/kg doses showing more favorable effects in cerebral and intestinal tissues than the 400 µg/kg dose (Figure 10).

## 3. Discussion

The present study provides evidence that LIRA attenuates multiorgan biochemical, histopathological, and immunohistochemical alterations in a TAA-induced rat model of hepatic encephalopathy. The observed effects included partial improvement in hepatic injury, normalization of serum and brain glutamine disturbances, attenuation of oxidative and inflammatory stress, a reduction in astroglial reactivity, and differential modulation of NRF2, MFN2, AKT1, mTOR, and VEGFA immunoreactivity across the liver, brain, and small intestine. These findings indicate that the effects of LIRA were not confined to a single tissue or molecular response but were accompanied by parallel hepatic, cerebral, and intestinal alterations. However, the responses were tissue-, marker-, and dose-specific rather than uniformly dose-dependent.

The TAA protocol used in the present study should be interpreted within the framework of acute liver failure-associated (Type A) HE rather than cirrhosis-associated chronic HE [28,29]. Short-course TAA administration is recognized in experimental HE guidelines and has been used in several studies to reproduce acute hepatic failure accompanied by hyperammonemia and cerebral metabolic, astroglial, and neurovascular alterations [28,29,30,31,32]. However, as with other toxin-based models, a direct contribution of TAA or its metabolites to cerebral alterations cannot be completely excluded [31]. Previous studies have detected TAA-related compounds in brain tissue after repeated administration, although their cerebral concentrations did not correlate with the stage of encephalopathy [31]. Therefore, the cerebral abnormalities observed in the present study should be interpreted as HE-associated alterations occurring in the context of TAA-induced acute hepatic injury, while recognizing that liver-mediated and potential direct toxin-associated cerebral effects cannot be fully distinguished by the present experimental design.

The extended post-TAA period in the present study was selected to evaluate the therapeutic effects of sustained LIRA treatment after the acute induction phase rather than to characterize only the immediate peak phase of encephalopathy. Approximately two-week LIRA regimens have been used in experimental studies evaluating sustained hepatic effects, including therapeutic administration after established TAA-induced liver disease [33,34]. Thus, the 14-day treatment period was intended to assess persistent post-injury multiorgan alterations and their response to LIRA. Importantly, this treatment duration should not be interpreted as evidence that overt encephalopathy persisted continuously throughout the entire post-TAA period.

The hepatic damage triggered by TAA represents a major upstream component of the pathological cascade leading to HE. In the present study, marked elevations in serum ALT and AST activities, together with severe hepatocellular necrosis and architectural disruption, indicated substantial hepatic injury, consistent with previous TAA-induced HE and liver injury models [9,35]. These alterations are consistent with TAA’s capacity to promote acute liver failure through reactive metabolite formation, oxidative damage, hepatocyte necrosis, and compromised hepatic metabolic function [10,36]. Although LIRA treatment produced numerical reductions in serum transaminase activities, particularly at 200 µg/kg, these changes were not statistically significant relative to the TAA group. Nevertheless, the accompanying improvement in hepatic histopathology suggests that the effects of LIRA were not confined to downstream cerebral alterations but also involved partial attenuation of the primary hepatic injury. Hepatoprotective effects of LIRA have previously been demonstrated in different experimental models of liver damage [17,37]. Preservation of hepatic tissue integrity may, in turn, mitigate the systemic metabolic and inflammatory burden contributing to HE progression, although this possibility was not directly assessed in the present study.

Failure of hepatic detoxification mechanisms is closely associated with disturbances in ammonia–glutamine metabolism linking hepatic injury to cerebral dysfunction [38]. In HE, astrocytic conversion of ammonia and glutamate to glutamine is initially compensatory; however, excessive glutamine accumulation may promote osmotic stress, mitochondrial dysfunction, and oxidative/nitrosative injury, thereby contributing to astrocyte swelling and neuronal damage [38,39]. Consistent with this framework, the concomitant elevation of serum and brain glutamine concentrations, degenerative neuronal changes, and increased GFAP immunoreactivity in TAA-treated rats suggests the development of an astrocyte-centered toxic metabolic response, as reported in experimental models of HE [7,9]. LIRA treatment reduced cerebral glutamine concentrations at all tested doses and attenuated neuronal degeneration and GFAP immunoreactivity. In serum, the most consistent normalization of glutamine concentrations was observed at 200 µg/kg. Notably, the 400 µg/kg dose reduced brain glutamine below control values, indicating a non-linear and potentially qualitatively different metabolic response rather than simple normalization. These findings are consistent with experimental evidence showing that LIRA can limit astroglial activation and neuroinflammatory injury in other models of cerebral metabolic and inflammatory stress [27,40]. Taken together, the parallel improvement in cerebral glutamine dysregulation, GFAP immunoreactivity, and neuronal morphology suggests that LIRA attenuated toxic metabolic processes associated with astroglial reactivity in TAA-induced HE.

A notable finding of this study was that fasting serum glucose concentrations remained unchanged across the experimental groups despite marked TAA-induced hepatic injury, consistent with the findings of Mohamed and Hafez [41]. Although hypoglycemia may accompany severe acute liver failure as a consequence of impaired hepatic glycogen storage and gluconeogenesis [42], the present findings indicate that altered circulating glucose is not an invariable metabolic feature of TAA-induced HE. The systemic glycemic response may instead vary according to the severity and temporal stage of hepatic injury, the TAA administration regimen, and other experimental conditions [41,43,44]. LIRA treatment did not significantly alter serum glucose concentrations at any tested dose. Given the glucose-dependent regulation of insulin and glucagon secretion by GLP-1 receptor agonists, together with their reported cytoprotective effects beyond glycemic control, the beneficial effects observed in this model appear to have occurred independently of major changes in systemic glucose concentrations [45].

Oxidative and nitrosative stress are important components of HE pathogenesis [4], and the present findings demonstrated marked tissue-dependent disturbances in redox homeostasis following TAA administration. TAA increased MDA levels in the liver, brain, and small intestine and reduced SOD activity in all three tissues. CAT activity was also decreased in the liver and small intestine, whereas cerebral CAT activity was not significantly altered. TAA-induced changes in NO metabolite levels were tissue-specific, with reductions in the liver, no significant alteration in the brain, and increases in the small intestine. LIRA partially ameliorated these abnormalities; however, its effects varied according to tissue, biochemical marker, and dose. Improvements were observed in several SOD and CAT responses and in selected MDA and NO metabolite alterations, whereas hepatic MDA, cerebral CAT, and intestinal NO metabolite levels were not significantly normalized. Thus, rather than producing uniform restoration of redox balance, LIRA exerted selective antioxidant and nitrosative stress-modulating effects, consistent with its previously reported cytoprotective actions [16]. The observed changes in Nrf2 immunoreactivity provide additional insight into the antioxidant responses associated with LIRA treatment. TAA markedly reduced Nrf2 immunoreactivity in the liver and brain, consistent with an alteration in Nrf2-associated antioxidant responses [46]. LIRA increased Nrf2 immunoreactivity in both tissues, with the most pronounced response observed at 200 µg/kg, in parallel with improvement in several oxidative stress parameters. This association supports a possible contribution of Nrf2-related cytoprotective responses to the hepatic and cerebral effects of LIRA. However, because Nrf2 nuclear translocation and downstream target gene expression were not assessed, these findings should be interpreted as changes in Nrf2-associated immunoreactivity rather than direct evidence of pathway activation. Moreover, although hepatic and cerebral alterations occurred concurrently in the present model, these parallel tissue responses should not be interpreted as direct evidence of liver-to-brain crosstalk, because circulating mediators and other mechanistic links between the two organs were not directly assessed.

MFN2 is a key regulator of mitochondrial fusion, mitochondrial dynamics, and endoplasmic reticulum–mitochondria communication, and alterations in MFN2 may impair cellular adaptation to oxidative and metabolic stress. Previous evidence has demonstrated that TAA-induced HE is accompanied by changes in MFN2 and other mitochondrial dynamics-related proteins, together with mitochondrial dysfunction and oxidative injury [11,47]. In the present study, TAA markedly reduced MFN2 immunoreactivity in both hepatic and cerebral tissues. These changes occurred alongside increased lipid peroxidation, impaired antioxidant enzyme responses, and inflammatory alterations, suggesting an association between altered MFN2 immunoreactivity and the redox–inflammatory disturbances accompanying TAA-induced HE. LIRA treatment differentially increased MFN2 immunoreactivity according to tissue and dose. In the liver, both 100 and 200 µg/kg restored MFN2 immunoreactivity to levels comparable with the control group, whereas the 400 µg/kg dose produced only a weak response. In the brain, the most pronounced restoration was observed at 200 µg/kg, while MFN2 immunoreactivity remained weak at 100 and 400 µg/kg. These findings indicate that LIRA differentially modulated MFN2 immunoreactivity, with the most consistent normalization observed at 200 µg/kg. This interpretation is broadly consistent with previous evidence showing that LIRA enhances mitochondrial respiration, ATP production, mitochondrial biogenesis, and bioenergetic resilience under metabolically and inflammatory stressful conditions [47]. However, because mitochondrial structure and function were not directly assessed, the observed increase in MFN2 immunoreactivity should not be interpreted as direct evidence of restored mitochondrial function.

TAA-induced alterations in AKT1 and mTOR immunoreactivity differed between hepatic and cerebral tissues. In the brain, TAA reduced both AKT1 and mTOR immunoreactivity, suggesting disruption of stress-responsive metabolic signaling during acute toxic metabolic injury. LIRA partially reversed these cerebral changes, with the most pronounced increases in both markers observed at 200 µg/kg. In the liver, however, the response was less uniform. Hepatic mTOR immunoreactivity was most clearly restored by LIRA at 200 µg/kg, whereas hepatic AKT1 immunoreactivity was not restored and remained weak at the 200 and 400 µg/kg doses. This divergence indicates that LIRA did not uniformly modulate all components of AKT1/mTOR-related responses across tissues. The present findings should also be interpreted in the context of conflicting evidence regarding AKT/mTOR signaling in experimental HE. Previous TAA-induced HE studies have reported increased PI3K/Akt/mTOR pathway activation rather than reduced immunoreactivity [10]. Such differences may reflect variations in disease duration, acute versus chronic injury, the tissue and cellular compartment examined, and, importantly, whether total AKT and mTOR abundance or their phosphorylated forms were assessed. Studies in other experimental models have also demonstrated that LIRA can modulate AKT/mTOR-related responses under hepatic and cerebral metabolic stress [17,18,19]. Accordingly, the observed changes in total AKT1 and mTOR immunoreactivity reflect alterations in the tissue expression of these proteins rather than direct evidence of AKT/mTOR pathway activation or restoration of pro-survival signaling.

A notable finding of the present study was the concomitant increase in VEGFA immunoreactivity in the liver, brain, and small intestine following TAA administration. This multiorgan pattern suggests that TAA-induced HE may involve vascular and angiogenic stress responses across multiple tissues. In the liver, increased VEGFA immunoreactivity may reflect a damage-associated angiogenic and tissue remodeling response, consistent with previous evidence linking hepatic VEGF alterations to TAA-induced fibrotic remodeling [48]. In the brain, increased VEGFA immunoreactivity may represent a neurovascular stress response occurring in association with astroglial activation, oxidative injury, and increased susceptibility of the blood–brain barrier [49]. The increase in intestinal VEGFA immunoreactivity is particularly noteworthy because it occurred in the absence of overt small intestinal histopathological damage. This dissociation may indicate an early or subhistological vascular stress response preceding detectable structural injury. However, because intestinal permeability, endothelial integrity, tight junction proteins, and mucosal inflammatory mediators were not evaluated, the present findings do not establish intestinal barrier dysfunction. LIRA differentially reduced VEGFA immunoreactivity according to tissue and dose. In the liver, all tested doses attenuated the TAA-induced increase, whereas the 100 and 200 µg/kg doses produced more favorable responses in cerebral and intestinal tissues than the 400 µg/kg dose. Although LIRA has been reported to enhance VEGF-related angiogenic responses in ischemic or reparative settings [50,51], it may exert different effects under inflammatory or endothelial stress conditions [52]. Accordingly, the reduction in VEGFA immunoreactivity observed in the present study may reflect context-dependent modulation of an exaggerated vascular stress response rather than generalized inhibition of angiogenesis.

LIRA exhibited a non-linear and endpoint-dependent dose–response profile in the present study. Although the 200 µg/kg dose produced the most consistent overall improvement across the evaluated parameters, the 100 µg/kg dose showed more favorable effects for selected endpoints, whereas the 400 µg/kg dose was frequently less effective. This variation may reflect tissue-specific sensitivity to GLP-1R stimulation, differences in receptor responsiveness, or high-dose pharmacodynamic effects, including alterations in food intake and energy balance that have been reported in experimental studies of LIRA. However, because body weight, food intake, pharmacokinetic exposure, and the effects of LIRA alone at the highest dose were not assessed, the reduced efficacy observed at 400 µg/kg cannot be attributed to a specific mechanism or interpreted as evidence of direct toxicity.

Several limitations should be considered. First, the absence of behavioral, cognitive, or neurological assessments precludes direct characterization of the functional severity and temporal persistence of HE. In addition, circulating ammonia concentrations were not measured; therefore, disturbances in ammonia metabolism were inferred primarily from serum and brain glutamine concentrations and accompanying cerebral alterations rather than directly demonstrated. Furthermore, because TAA is a systemically administered toxicant, a direct contribution of TAA or its metabolites to the observed cerebral alterations cannot be excluded. The present design also does not establish that overt encephalopathy persisted continuously throughout the 14-day treatment period. Second, although intestinal redox abnormalities and VEGFA immunoreactivity suggest that intestinal responses may accompany the hepatic and cerebral alterations, microbiota composition, intestinal permeability, tight junction proteins, endotoxin translocation, and mucosal inflammatory mediators were not assessed; therefore, the contribution of gut-derived signals and intestinal barrier dysfunction remains inferential. Third, Nrf2, MFN2, AKT1, mTOR, GFAP, and VEGFA were evaluated using semiquantitative immunohistochemistry. Although this approach demonstrated tissue-level changes in immunoreactivity, it did not establish pathway activity, cell-specific localization, phosphorylation status, or causal interactions. The molecular findings of the present study were based on a limited number of biochemical and semiquantitative immunohistochemical markers. Therefore, the observed changes should be interpreted as associative tissue-level responses rather than as evidence that the corresponding molecular pathways causally mediated the effects of LIRA. In particular, the absence of phosphorylation-based analyses, nuclear Nrf2 assessment, quantitative protein validation, and direct mitochondrial structural or functional measurements limits mechanistic interpretation. Furthermore, the histopathological and immunohistochemical evaluations were semiquantitative, and the IHC findings were not independently validated using Western blotting, quantitative protein analysis, or other complementary molecular techniques. Therefore, these findings should be interpreted as tissue-level patterns of immunoreactivity rather than definitive quantitative evidence of protein expression or molecular pathway activity. Fourth, the glucose findings cannot be attributed to a defined metabolic mechanism because insulin, glucagon, hepatic glycogen content, and food intake were not determined. Although the experimental design was developed with consideration of the Reduction principle of the 3Rs to minimize animal use, the absence of a LIRA-only group limits assessment of the basal and dose-specific effects of LIRA and restricts interpretation of the non-linear responses observed at the highest dose. An additional limitation concerns the characterization of the HE phenotype and liver–brain interaction. Although TAA-treated animals exhibited persistent hepatic injury together with increased serum and cerebral glutamine concentrations, cerebral histopathological alterations, and increased GFAP immunoreactivity, circulating ammonia concentrations, neurological or behavioral HE scores, hepatic synthetic-function parameters, and direct measures of liver-to-brain signaling were not assessed. Therefore, the severity of encephalopathy and direct functional crosstalk between the liver and brain cannot be established from the present data. The cerebral findings should consequently be interpreted as HE-associated alterations occurring in the context of TAA-induced hepatic injury rather than as direct evidence of a demonstrated liver–brain axis. Future studies integrating functional testing, microbiome and barrier analyses, molecular pathway validation, and chronic or sex-inclusive HE models are therefore required to more precisely define the translational relevance of LIRA.

## 4. Materials and Methods

### 4.1. Animals and Ethical Approval

All animal experiments were conducted in accordance with relevant guidelines for the care and use of laboratory animals and were reported in compliance with the ARRIVE guidelines [53]. The experimental protocol was reviewed and approved by the Animal Experiments Local Ethics Committee of Erzincan Binali Yildirim University (29 January 2026, decision no: 10). A total of 30 adult male Sprague–Dawley rats weighing 220 ± 20 g were obtained from the Experimental Animals Application and Research Center of Erzincan Binali Yildirim University (DEHAM, Erzincan, Türkiye). The animals were housed in standard plastic cages under controlled laboratory conditions at a temperature of 22 ± 2 °C, relative humidity of 40–60%, and a 12 h light/12 h dark cycle. Standard pellet chow and tap water were provided ad libitum. Before the experimental procedures, the animals were acclimatized to the laboratory conditions for 7 days. All efforts were made to minimize animal suffering and reduce the number of animals used.

### 4.2. Experimental Design

Following the acclimatization period, the animals were randomly allocated into five groups, with six rats in each group, as follows:

Control group (*n* = 6): Rats received normal saline intraperitoneally (i.p.) on days 1, 2, and 3. Beginning on day 4, they received normal saline subcutaneously (s.c.) once daily for 14 consecutive days.

TAA group (*n* = 6): Rats received Thioacetamide (TAA; ≥99%, Cat. No. 163678; Sigma-Aldrich, St. Louis, MO, USA) (TAA; 200 mg/kg, i.p.) on days 1, 2, and 3. Beginning on day 4, they received normal saline s.c. once daily for 14 consecutive days.

TAA+LIRA groups (*n* = 6 per group): Rats received TAA (200 mg/kg, i.p.) on days 1, 2, and 3. Beginning on day 4, they were treated with Liraglutide (Saxenda^®^; 6 mg/mL; Novo Nordisk A/S, Bagsværd, Denmark) at doses of 100, 200, or 400 µg/kg/day s.c. for 14 consecutive days.

The doses and administration schedules of TAA and LIRA were selected based on previous studies [35,54]. To minimize the risk of TAA-associated renal dysfunction, hypoglycemia, and electrolyte imbalance, all TAA-exposed rats received 0.5 mL of lactated Ringer’s solution containing 10% dextrose sc at 12 h intervals following each TAA injection [36].

### 4.3. Sample Collection

At the end of the experimental period, 24 h after the final LIRA or saline administration on day 18, animals were fasted for 8 h before terminal blood collection, with free access to water, to minimize the influence of recent food intake on circulating glucose concentrations [55]. Rats were anesthetized with thiopental sodium (30 mg/kg, i.p.), and blood samples were collected by intracardiac puncture. Blood samples were centrifuged at 1200 rpm for 10 min at 4 °C, and the resulting serum samples were aliquoted and stored at −80 °C until biochemical analysis. Following this, the liver, brain, and small intestinal tissues were rapidly excised and rinsed with ice-cold phosphate-buffered saline (PBS, pH 7.4). Portions of the liver, brain, and small intestinal tissues were fixed in 10% neutral-buffered formalin for histopathological and immunohistochemical analyses. The remaining tissue samples were processed for biochemical measurements.

For biochemical analyses, liver, small intestinal, and brain tissues were homogenized in ice-cold phosphate-buffered saline (PBS; 50 mM, pH 7.4) at a ratio of 50 mg tissue to 2 mL buffer. The homogenates were centrifuged at 5000× *g* for 15 min at 4 °C, and the resulting supernatants were carefully collected and stored at −80 °C until analysis. The supernatants were used for the determination of oxidative stress parameters, antioxidant enzyme activities, and brain glutamine levels. Total protein concentrations were determined using the Bradford method, and enzyme activities and tissue biochemical parameters were normalized to protein content where applicable [56]. Biochemical measurements were conducted at the Research and Development Laboratory, Faculty of Medicine, Erzincan Binali Yıldırım University, Erzincan, Türkiye.

### 4.4. Biochemical Parameters

Serum levels of alanine aminotransferase (ALT) and aspartate aminotransferase (AST) were determined with an automated AU5800 Chemistry Analyzer (Beckman Coulter, Brea, CA, USA). Analyses were performed based on kinetic UV ultraviolet methods recommended by the International Federation of Clinical Chemistry and Laboratory Medicine (IFCC). The assays were based on monitoring the rate of nicotinamide adenine dinucleotide (NADH) oxidation at 340 nm during coupled enzymatic reactions, and the reduction in absorbance was directly proportional to the enzyme activities.

Serum and brain tissue glutamine levels were determined using a commercial ELISA kit (Cat. No. CK-bio-27615; Shanghai Coon Koon Biotech Co., Shanghai, China) in accordance with the manufacturer’s protocol. According to the manufacturer’s specifications, the intra-assay coefficient of variation (CV) was <10%, the inter-assay CV was <12%, and the assay sensitivity was 2 ng/mL.

Serum glucose concentrations were evaluated using an enzymatic colorimetric method based on the glucose oxidase–peroxidase reaction on an automated chemistry analyzer. In this method, glucose is oxidized by glucose oxidase to gluconic acid and hydrogen peroxide (H_2_O_2_). Subsequently, H_2_O_2_ reacts with a chromogenic substrate in the presence of peroxidase, generating a colored compound whose absorbance is determined spectrophotometrically. The intensity of the color formed is directly proportional to the glucose concentration in the sample. All measurements were performed in duplicate, and quality control sera with low and high glucose concentrations were analyzed concurrently to ensure analytical accuracy and precision. Glucose concentrations were expressed as mg/dL.

### 4.5. Histopathological Examination

Following necropsy, brain, liver, and small intestinal tissues were excised and fixed in 10% neutral buffered formalin. After routine tissue processing through graded alcohols and xylene, samples were embedded in paraffin. Sections measuring 4 µm were obtained with a rotary microtome, mounted on glass slides, and stained with hematoxylin and eosin (H&E) for routine histopathological evaluation.

All rats were included in the histopathological evaluation. Six sections from each tissue were examined per animal, and six randomly selected non-overlapping microscopic fields were assessed in each section. Hepatic sections were evaluated for hepatocellular degeneration, necrosis, and disruption of tissue architecture. Cerebral sections were evaluated for pyknotic and degenerative neuronal alterations, whereas small intestinal sections were assessed for epithelial degeneration, villus disruption, and inflammatory or structural alterations. Sections were graded for degenerative and necrotic alterations as follows: absent, mild (5–10%), moderate (11–20%), and severe (>21%) [57]. Field-level scores were combined to obtain a single representative score for each tissue from each animal, and animal-level scores were used for statistical analysis. Histopathological evaluation was performed by an experienced pathologist blinded to the experimental groups.

### 4.6. Oxidative Stress and Antioxidant Biomarkers in Hepatic, Intestine and Brain Tissues

Malondialdehyde (MDA) levels in liver, small intestinal, and brain tissue homogenates were determined according to the method described by Ohkawa et al. [58], with modifications introduced by Kurt et al. [59]. The assay is based on the reaction of MDA with thiobarbituric acid (TBA), forming a pink-colored thiobarbituric acid reactive substances (TBARS) complex that can be quantified spectrophotometrically at 532 nm. Briefly, 25 µL of tissue homogenate was mixed with 25 µL of sodium dodecyl sulfate (80 mg/mL) and 1 mL of reaction mixture containing 20 g/L acetic acid, 1.06 g thiobarbituric acid, and 180 mL distilled water in a 1.5 mL microcentrifuge tube. The reaction mixture was incubated at 95 °C for 60 min and subsequently cooled to room temperature. After cooling, the samples were centrifuged at 1200× *g* for 10 min, and the absorbance of the supernatant was measured at 532 nm using a spectrophotometer. MDA concentrations were calculated from a standard curve generated using 1,1,3,3-tetramethoxypropane and expressed as nmol/g tissue.

Superoxide dismutase (SOD) activity in hepatic, small intestinal, and brain tissues was measured according to the method of Sun et al., which assesses inhibition of nitroblue tetrazolium reduction by superoxide radicals produced via the xanthine–xanthine oxidase system. The absorbance was recorded at 560 nm, and SOD activity was normalized relative to protein concentration [60].

Catalase (CAT) activity in all tissue homogenates was determined using the ammonium molybdate method described by Goth [61]. This method is based on the enzymatic decomposition of hydrogen peroxide (H_2_O_2_) into water and oxygen by catalase. Briefly, tissue homogenates were incubated with 65 mM H_2_O_2_ prepared in an appropriate buffer for a specified reaction period. The reaction was then terminated by adding an ammonium molybdate solution. Residual H_2_O_2_ reacted with ammonium molybdate to form a stable yellow-colored complex, and its absorbance was measured at 405 nm using a microplate reader. Catalase activity was calculated from the difference between the absorbance values of the sample and control reactions.

Myeloperoxidase (MPO) activity in tissue samples was determined using the colorimetric spectrophotometric method described by Bradley et al. [62]. Briefly, tissue homogenates were centrifuged, and the resulting supernatants were added to the reaction mixture containing 0.53 mM o-dianisidine dihydrochloride and 0.0005% hydrogen peroxide (H_2_O_2_). The reaction mixtures were incubated at 37 °C for 5 min, and the formation of the orange-colored oxidation product was monitored by measuring the absorbance at 450 nm using a microplate reader. MPO activity was calculated from the change in absorbance, normalized to total protein concentration, and expressed as U/mg protein.

Nitric oxide (NO) production in tissue samples was assessed using a Cayman Chemical Nitrate/Nitrite Colorimetric Assay Kit (Item No: 780001) according to the manufacturer’s instructions. Since NO is highly unstable and rapidly reacts under laboratory conditions, the assay quantifies its stable metabolites, nitrite (NO_2_^−^) and nitrate (NO_3_^−^). For total nitrate/nitrite measurement, nitrate in the samples was first converted to nitrite by nitrate reductase supplied in the kit. The resulting nitrite was then quantified using the Griess reaction, and absorbance was measured at 540 nm using a microplate reader. NO metabolite concentrations were calculated from nitrite and nitrate standard curves provided with the kit and normalized to total protein concentration, and expressed as nmol/mg protein.

### 4.7. Immunohistochemical Analysis

Immunohistochemical staining was performed to assess tissue expression of Nrf2, Mfn2, AKT1, mTOR, VEGFA, and GFAP. Liver sections were immunostained for MFN2, AKT1, mTOR, VEGFA, and Nrf2, whereas brain sections were additionally evaluated for GFAP immunoreactivity. Intestinal sections were examined only for VEGFA immunoreactivity. Briefly, 4 μm thick paraffin sections on poly-L-lysine-coated slides were deparaffinized in xylene and rehydrated through a graded alcohol series. Following washing with PBS, endogenous peroxidase activity was blocked by incubation with 3% H_2_O_2_ for 10 min. Antigen retrieval was performed by heating sections twice for 5 min at 500 W in an antigen retrieval solution. The sections were then incubated overnight at 4 °C with the following primary antibodies, each diluted 1:200: Mfn2 (Bioassay Technology Laboratory, BT-Lab, Shanghai, China; Cat. No. BT-AP05382), AKT1 (Santa Cruz Biotechnology, Dallas, TX, USA; Cat. No. sc-5298), mTOR (Santa Cruz Biotechnology, Dallas, TX, USA; Cat. No. sc-517464), VEGFA (Affinity Biosciences, Cincinnati, OH, USA; Cat. No. AF5131), Nrf2 (BT-Lab, Shanghai, China; Cat. No. BT-AP12024), and GFAP (BT-Lab, Shanghai, China; Cat. No. BT-AP03549).

Following incubation with the primary antibodies, the sections were processed with a Large Volume Detection System: anti-Polyvalent, HRP secondary detection kit (HRP (Cat. No. TP-125-HL; Thermo Fisher Scientific/Lab Vision, Waltham, MA, USA) as recommended by the manufacturer’s protocol. Immunoreactivity was visualized using 3,3′-diaminobenzidine as the chromogen. The sections were counterstained with Mayer’s hematoxylin, mounted with an aqueous mounting medium, and evaluated using a light microscope. Immunohistochemical staining intensity was evaluated semiquantitatively in six randomly selected, non-overlapping microscopic fields per section using the following ordinal scale: absent (−), mild (+), moderate (++), severe (+++), or very severe (++++).

### 4.8. Statistical Analysis

Statistical analyses were performed using IBM SPSS version 22.0 for Windows, (IBM Corp., Armonk, NY, USA). The normality of continuous variables was assessed using the Shapiro–Wilk test, and the homogeneity of variances was evaluated using Levene’s test. Normally distributed data with homogeneous variances were analyzed using one-way analysis of variance (ANOVA), followed by Tukey’s honestly significant difference post hoc test. For normally distributed data with unequal variances, Welch’s one-way ANOVA followed by the Games–Howell post hoc test was used. Non-normally distributed continuous variables were analyzed using the Kruskal–Wallis test, followed by Bonferroni-adjusted pairwise comparisons.

Histopathological and immunohistochemical scores were treated as ordinal variables and analyzed using the Kruskal–Wallis test. The individual animal was considered the experimental unit, and animal-level values were used in all statistical analyses. Continuous data are presented as the mean ± standard deviation (SD), whereas ordinal histopathological and immunohistochemical scores are presented as the median and interquartile range. A two-sided *p* value < 0.05 was considered statistically significant.

The data generated and analyzed during the current study are available from the corresponding author upon reasonable request.

Generative artificial intelligence was used only for language editing and stylistic refinement of the manuscript. It was not used for study design, data analysis, result interpretation, or generation of scientific content.

## 5. Conclusions

To our knowledge, this study provides initial evidence regarding the effects of LIRA in a TAA-based model of acute liver injury with HE-associated cerebral alterations. LIRA partially ameliorated hepatic and cerebral structural injury, reduced serum and brain glutamine disturbances, attenuated selected oxidative, nitrosative, and inflammatory alterations, and decreased cerebral astroglial reactivity. These effects were accompanied by tissue- and dose-specific modulation of Nrf2, MFN2, AKT1, mTOR, and VEGFA immunoreactivity across the liver, brain, and small intestine. The responses were non-linear and endpoint-dependent, with the 200 µg/kg dose producing the most consistent overall improvement and the 100 µg/kg dose showing more favorable effects for selected parameters. Collectively, these findings suggest that LIRA may alleviate multiorgan tissue stress related to TAA induced acute hepatic injury and HE-associated cerebral alterations. Further studies incorporating functional neurological assessment, direct ammonia measurement, quantitative molecular validation, and chronic or clinically relevant HE models are required to clarify its mechanisms and translational potential.

## Data Availability

The original contributions presented in this study are included in the article. Further inquiries can be directed to the corresponding authors.

## Abbreviations

The following abbreviations are used in this manuscript:

HE	Hepatic encephalopathy
TAA	Thioacetamide
LIRA	Liraglutide
GLP-1R	Glucagon-like peptide-1 receptor
ALT	Alanine aminotransferase
AST	Aspartate aminotransferase
MDA	Malondialdehyde
SOD	Superoxide dismutase
CAT	Catalase
MPO	Myeloperoxidase
NO	Nitric oxide
Nrf2	Nuclear factor erythroid 2-related factor 2
MFN2	Mitofusin-2
AKT1	AKT serine/threonine kinase 1
mTOR	Mechanistic target of rapamycin
GFAP	Glial fibrillary acidic protein
VEGFA	Vascular endothelial growth factor A
BBB	Blood–brain barrier
ROS	Reactive oxygen species
LPO	Lipid peroxidation
IHC	Immunohistochemistry
H&E	Hematoxylin and eosin
PBS	Phosphate-buffered saline
DAB	3,3′-Diaminobenzidine
